# The effects of allostatic load on patients undergoing surgery: a systematic review

**DOI:** 10.3389/fsurg.2026.1866869

**Published:** 2026-08-21

**Authors:** Whitaker Reid, Nidhi Rhea Udyavar

**Affiliations:** 1 Department of Medical Education, Burnett School of Medicine at TCU, Fort Worth, TX, United States; 2 Department of Surgery, University of Washington, Seattle, WA, United States

**Keywords:** allostatic load, chronic stress, postoperative outcomes, socioenvironmental stressors, surgery, surgical oncology

## Abstract

**Introduction:**

Allostatic load (AL) is a measure of physiologic adaptation to repetitive environmental stressors and is associated with poor health outcomes and chronic conditions, ranging from cardiovascular disease and type 2 diabetes to malignancy. Given the acute rise in physiologic stress that accompanies surgically induced trauma, AL has the potential to affect surgical outcomes, particularly for the invasive, complex, lengthy procedures common in surgical oncology. The aims of this study were to systemically review existing literature surrounding AL and surgery, and to contextualize these findings accordingly.

**Methods:**

A systematic review was conducted in May 2024 via PubMed, Embase, Cochrane, Web of Science, and PsycINFO to identify existing literature on AL and surgery. Study quality and sources of bias were assessed via the Mixed Methods Appraisal Tool. Data from original studies were extracted and compiled, and a similar process was repeated for related review articles.

**Results:**

A total of 10 original research studies explored the relationship between AL and surgical topics. Eight of these studies attempted to quantify AL as either a numerical composite index or via single or multiple representative biomarkers. AL emerged as (1) a specific predictor of surgical outcomes and (2) an explanatory mechanism for racial and socioeconomic disparities in surgical populations, as AL is known to be increased in socially disadvantaged individuals. Four of 10 included studies explored surgical oncology populations exclusively.

**Discussion:**

AL appears to impact surgical care and demonstrates the potential to not only predict postoperative outcomes (i.e., postoperative complications) but also identify high-risk surgical patients. This relationship appears to be most well-defined in surgical oncology relative to any other surgical specialty. By illuminating the connections between AL and surgery, targeted interventions aimed at reducing AL may be implemented to mitigate postoperative complications and improve the quality of surgical care. Future work in this area should include prospective studies examining preoperative AL, specific postoperative outcomes, and the impact of personalized AL-centered interventions on adverse perioperative outcomes.

## Introduction

Over the last fifteen years, the concept of “allostatic load” (AL) and its relevance to a variety of health outcomes have gained significant traction in clinical research. AL is defined as the physiologic adaptation to cumulative situational, environmental, psychological, and physical stress (1). Similar to the phenomenon of epigenetics, wherein environmental factors can directly alter gene expression among individuals across multiple generations, AL is also based upon the principle that physiologic stress triggers adaptive changes as the body attempts to restore homeostasis in response to repetitive stressors. AL reflects the cumulative effect of daily life experiences that are considered routine but are subjectively experienced by the individual as a stressor, as well as major challenges (i.e., life events). The physiological consequences of certain health-damaging behaviors may be a reaction to these stressors, such as poor sleep, smoking, disordered alcohol use, illicit drug use, and poor dietary habits, which then further overload allostatic mechanisms. This effort to adapt to chronic stressors leads to derangements across multiple physiologic systems, which then contribute to adverse clinical outcomes such as cardiovascular diseases, type 2 diabetes, early menarche, and breast and ovarian cancers (2, 3).

Surgery presents a significant acute physiologic stressor to any individual, regardless of baseline AL, and the observed variability in tolerance and restoration of homeostasis in the perioperative setting may be driven in part by AL. Although there has been a notable expansion of knowledge surrounding AL and its association with various clinical entities such as cardiovascular disease, metabolic syndrome, and even cancer risk, much remains undefined in the relationship between AL and surgical outcomes. The acute response to surgical trauma is characterized by hypermetabolism and inflammation, which may be either attenuated or augmented by the presence of chronic physiologic stress. While this immediate effect is necessary to facilitate healing from tissue injury, chronic stress as represented by AL may lead to dysregulation of the systems that modulate this response. Acute surgical stress and its associated immunomodulatory and hormonal effects have even been linked to an increased risk of cancer progression and metastasis—an association that is also observed among patients with higher AL (4–7). AL has also been connected to failure of immunotherapy for cancer treatment, as well as increased cancer-related mortality (8, 9). This is particularly relevant to surgical oncologists, as the procedures that they perform are often invasive, complex, and lengthy—factors that are associated with a more pronounced surgical stress response. Therefore, it is necessary to explore the relationship among surgery and the associated acute stress response, AL as a measure of chronic stress, and surgical outcomes.

AL is often operationalized as a composite index of various laboratory biomarkers and physical examination measurements, intended to represent multiple organ systems. Commonly used biomarkers include high-density lipoprotein (HDL) cholesterol, total cholesterol, HbA1c, cortisol, C-reactive peptide (CRP), and serum albumin; many of these are also used to measure the acute surgical stress response, highlighting the synergistic effects of acute and chronic stress on physiology (10). Waist circumference, systolic/diastolic blood pressure, and resting heart rate are also included in many versions of the AL index (11). Critically, most of these markers and measurements are easy to collect in the perioperative setting.

While “stress” is a universal experience that all individuals will face to some extent, certain individuals are subjected to the necessary development of biological adaptations to repetitive stressors that may be pathologic. AL is inherently linked to the lived experiences that are specific to an individual's unique combination of socioeconomic factors, race and ethnicity, built environment, gender identity, and social support (12–14). Socially vulnerable patients have been shown to have higher AL, as well as increased risk of postoperative complications and increased postoperative length of stay (12, 15, 16). Racially minoritized patients also experience much higher rates of cancer-specific mortality and surgical complications. Because racial and socioeconomic disparities persist across multiple domains, including surgical care, AL is becoming increasingly germane to our understanding of the specific etiologies of these health inequities and how we can more effectively address them through targeted, patient-level interventions that venture beyond the existing paradigm (17, 18).

Introducing the stress of surgical trauma may exacerbate the physiologic abnormalities caused by increased AL, or baseline AL may blunt the necessary acute surgical stress response. Either scenario would affect surgical outcomes, particularly among the most invasive and complex procedures associated with high surgical stress, such as surgical oncology. With approximately 300 million surgeries occurring globally in a single year and a projected growth from 9 million in 2018 to 13.8 million in 2040 for cancer-related operations, specifically, the necessity and potential benefit of establishing a connection between AL and surgical outcomes are substantial (19, 20). As such, the aims of this study were to systemically review the literature surrounding AL and surgery, synthesize these findings accordingly, and identify areas of opportunity for future research.

## Methods

We conducted a systematic review of the literature investigating AL in relation to surgical operations or related topics (i.e., wound healing and pain). This review adhered to PRISMA guidelines (21). Multiple core databases were used, and search terms were refined to encompass all variant terminology. Specifically, PubMed, Embase, Cochrane, Web of Science, and PsycINFO were searched in May 2024 with no date limits to minimize the possibility of selection bias. The following search was constructed for PubMed utilizing medical subject headings (MeSH) and keywords and was subsequently translated to meet formatting requirements of the alternative databases: (“Allostasis”[MeSH] OR “allosta*”[tiab] OR “Socioenvironmental stressor*”[tiab] OR “Socioenvironmental factor*”[tiab]) AND (“Surgical Procedures, Operative”[MeSH] OR “Postoperative Complications"[MeSH] OR “surgi”[tiab] OR “surger*”[tiab] OR “postsurg*”[tiab] OR “post-surg*”[tiab] OR “postoperative*”[tiab] OR “post-operative*”[tiab]). To maximize the scope of literature identified through our search, truncation (“allosta*”) was utilized to capture possible variants (allostasis, allostatic overload, etc.) of our search terms. Translations of this search query across the 4 other literature databases are listed in Supplementary Table 1, which were constructed in consultation with a senior-level university librarian.

In order to further augment the comprehensiveness of our search and minimize the potential of selection bias, we also searched for relevant grey literature. Within the original search strategy as described above, results from Embase included conference materials and PsychINFO yielded dissertations. The Web of Science search string was repeated utilizing the Web of Science ProQuest Dissertations and Theses Citation Index. Preprints were searched in Europe PMC. In effort to identify reports and white papers, the American Psychological Association, American Medical Association, Society of Surgical Oncology, American Thyroid Association, and American Association of Endocrine Surgeons websites were queried utilizing simple searches, such as “sociological factors surgery” or “allostatic load”.

**Figure 1:**
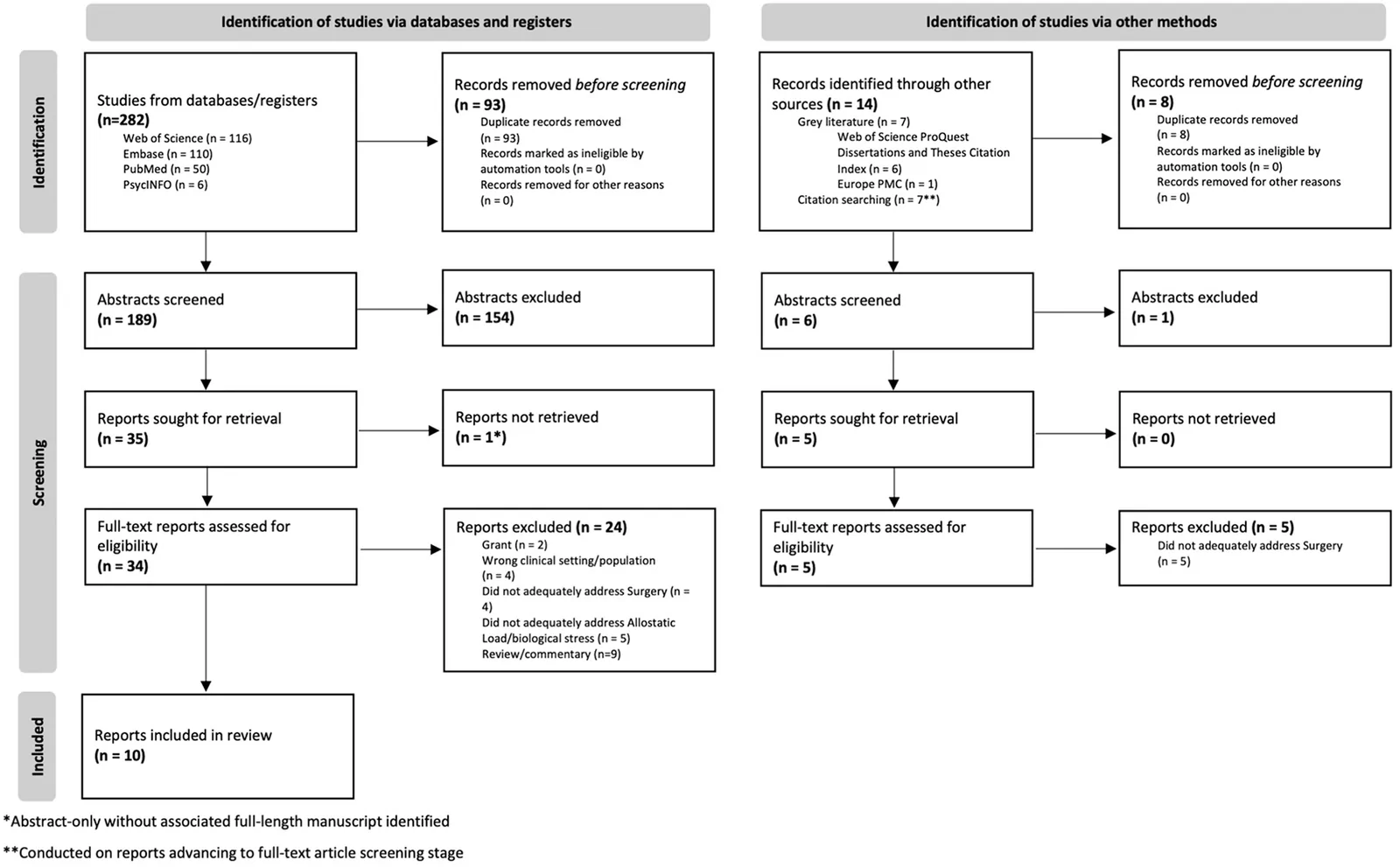
PRISMA flow diagram. Adapted from Page MJ, et al. BMJ 2021;372:n71. doi: 10.1136/bmj.n71. CC BY 4.0.

Inclusion criteria were articles with a primary focus of both AL and surgery (including major or minor procedures) and written in the English language. Due the shared human experience of stress worldwide, the global utilization and access to surgical procedures, and the nascency of this area of research, articles were not limited to being published within the US. We excluded animal studies or those focused on pediatric populations. No unpublished or original data were incorporated into this review. Search results were imported into Covidence, which was utilized for article screening, data extraction, and generation of the PRISMA flow diagram outlining each phase of publication assessment within this systematic review (Figure 1).

Article screening was conducted by both authors (WR, RU), with any discrepancies prompting joint discussion to inform a final disposition. Following the removal of duplicate texts, title/abstract screening was initiated, followed by full-text review of the studies deemed potentially relevant. Publications reaching the full-text stage were assessed for both topic relevance and potential sources of bias. The reference lists of the studies reaching this stage also underwent review, with potentially relevant citations also undergoing screening as illustrated in the same PRISMA flow diagram (Figure 1). Study quality and bias were measured via the Mixed Methods Appraisal Tool (MMAT), version 2018 (Supplementary Table 2) (22). Results from this assessment were documented accordingly, with a composite score generated upon deliberation between the authors (WR, RU). Publications that did not adequately address allostatic load/biological stress or surgical topics, contained only expert opinion without original data, or were either systematic reviews or grant proposals were excluded from full-text review. Relevant reviews and author commentaries were compiled in Supplementary Table 3 and briefly noted in the Results below for additional context.

## Results

As shown in Figure 1, a total of 282 studies were identified across Embase, PsycINFO, PubMed, and Web of Science. An additional 14 studies were found as grey literature or through reference list screening. Of these 296 combined reports, 101 duplicates were excluded. The subsequent 195 publications were respectively assessed for eligibility based on the inclusion and exclusion criteria defined above. Ultimately, 155 and 29 studies were excluded via title/abstract and full-text review, respectively. One study was removed during article retrieval as no associated manuscript was identified. The resultant 10 publications underwent in-depth evaluation with subsequent data extraction (Figure 1) (23–32). Study characteristics, methodology, and findings were outlined in Table 1.

**Table 1:** Data extraction from original research in allostatic load and surgery.

Authorship	Study population		Description & methodology					Allostatic load findings
	Population	Race/Ethnicity	Study design	Data source	AL Measures	Intervention/Exposure	Outcome	
Abshire et al., 2018 (23)	Adults ages 21+ ≥ 3 months post-LVAD implantation at a single institution (*n* = 44)	45% White, 43% Black, 11% other	Cross-sectional comparative	Single institution cohort	Yes	LVAD implant strategy	Physiologic/psychologic stress QoL/functional status	Cortisol, CRP, and sleep quality did not differ by implant strategy (except waking cortisol level; significantly higher in destination therapy group) There were no significant differences in perceived stress, depression, or fatigue by implant strategy Normal cortisol awakening response was associated with low levels of depressive symptoms Higher salivary cortisol AUC levels were associated with improved functional status but not QoL Decreased sleep quality and higher psychological stress response were associated with lower QoL
Becker & Granzotti, 2019 (24)	Patients post-CABG in 1998–2015 (*n* = 5,032,985)	Not specified	Retrospective cohort	HCUP-NIS	No	Gender Race/ethnicity	In-hospital CABG mortality rates	Females and Black males have higher in-hospital CABG mortality rates, possibly explained by AL, even though AL represents long-term adaptation to chronic stress Targeting AL among Black patients earlier in life could reduce racial disparities in mortality Cites Chyu & Upchurch (11) for calculating AL using 10 biomarkers spanning cardiovascular, inflammatory, and metabolic systems
Chen et al., 2024 (25)	Women ages 18 + diagnosed with stage I-III BC at a single institution (*n* = 4,089)	87% White, 9% Black, 4% other	Retrospective cohort	Institutional Cancer Registry	Yes	Neighborhood opportunity (via OOI)	AL All-cause mortality	Low OOI was associated with greater risk for all-cause mortality in a dose-dependent relationship Low neighborhood opportunity was associated with greater odds of high AL even in fully adjusted models Domain-specific adjusted analyses demonstrated significant associations between both low educational attainment and poor housing for higher AL Although mortality risk did not increase with high AL in patients living in higher-opportunity environments, mortality risk was greater among patients with high AL residing in lower-opportunity areas
Chen et al., 2024 (26)	Women ages 18 + diagnosed with stage I-III BC at a single institution (*n* = 4,296)	87% White, 9% Black, 4% other	Retrospective cohort	Institutional Cancer Registry	Yes	Racialized economic segregation (via ICE)	AL All-cause mortality	Living in neighborhoods with high racialized economic segregation was associated with greater risk of all-cause mortality in fully adjusted models Neighborhood racialized economic segregation was significantly associated with AL in a non-linear dose-response relationship
Chen et al., 2024 (27)	Women ages 18 + diagnosed with stage I-III BC at a single institution (*n* = 4,459)	87% White, 9% Black, 4% other	Retrospective cohort	Institutional Cancer Registry	Yes	AL	Postoperative complications	Patients with high AL had significantly higher risk of POC in adjusted analysis Identified linear dose-dependent relationship between increasing AL and POC development Albumin was the primary biomarker associated with POCs
Garcia et al., 2021 (28)	Females ages 18 + with CMI (*n* = 62)	89% White, 3% Black, 5% other (from *n* = 44)	Cross-sectional comparative	Participants recruited online	Yes	CMI surgical status Loneliness Disability	Stress/resilience biomarkers	Identified loneliness as the most important predictor of stress/resilience biomarkers, but only among these surgical patients Within the surgically decompressed group, higher levels of loneliness were associated with increased cortisol (stress marker) and lower estrogen (resilience marker) Suggests that loneliness triggers stress response
Hong & Lee, 2022 (29)	Patients post-thyroidectomy with routine follow-up at a single institution (*n* = 106)	Not specified	Prospective cohort	Enrolled patients	Yes	Stress (via SOSS, KDSI, SRRS, and OQ)	TSH	TSH had a significant relationship with stress using a modified SOSS questionnaire but not the KDSI, standard SOSS, and SRRS Based on the modified SOSS, TSH levels increased with the occurrence of a stressful event Although TSH has the potential as a stress marker, TFTs demonstrate variable patterns in response to AL in patients with functioning thyroid
Hughes et al., 2022 (30)	Patients ages 40 + post-revascularization for severe PAD in 2010–2014 (*n* = 131,529)	Not specified	Retrospective cohort	Nationwide Readmissions Database	No	SES (via MHI by ZIP code)	Lower extremity reconstruction outcomes	MHI was inversely related to amputation following initial surgical revascularization on adjusted analysis 4th (vs. 1st) MHI quartile patients had significantly lower 30-day readmission rates Chronic biologic adaptation to environmental stress (i.e., AL, biology of poverty) discussed as potential driver for disparity in PAD severity
Irelli et al., 2022 (31)	Female patients with BC undergoing chemotherapy at a single institution (*n* = 61)	Not specified	Cross-sectional comparative	Enrolled patients	Yes	Physical impairment (via CIRS) Pain perception Age	Psychological distress	Increased physical symptoms and pain were associated with worse psychological outcomes Physical impairment (high comorbidity) appeared to be predictive of negative emotional adaptation There were no significant differences in psychological outcomes by type of surgical intervention (lumpectomy vs. mastectomy) Suggests that physical impairment as an AL factor could be routinely incorporated as a screening tool to inform tailored effective psychological support
McGuinness et al., 2018 (32)	Paired preperfusion and postperfusion renal biopsies from deceased-donor kidneys subsequently transplanted (*n* = 55)	98.2% Caucasian, 1.8% Asian	Prospective cohort	Renal biopsies & enrolled donor recipients	Yes	Allograft AL (via molecular signatures)	Graft function (via serum Cr, eGFR)	BioAge in preperfusion renal biopsies correlated with DGF and long-term post-transplant allograft function Identified 42 molecular transcript signatures associated with impaired allograft function, consistent with elevated AL

AL, llostatic load; BC, breast cancer; CIRS, cumulative illness rating scale; CMI, chiari malformation type I; Cr, creatinine; DGF, delayed graft function; eGFR, estimated glomerular filtration rate; HCUP-NIS, healthcare utilization project-nationwide inpatient sample; ICE, index of concentration at the extremes; KDSI, Korean version of the daily stress inventory; LVAD, left ventricular assist device; MMAT, mixed methods appraisal tool; OOI, ohio opportunity index; OQ, open-ended questionnaire; PAD, peripheral artery disease; POC, postoperative complication; QoL, quality of life; SOSS, stress overload scale-short; SRRS, social readjustment rating scale; TSH, thyroid-stimulating hormone.

Quality and bias assessment using the MMAT indicated that all 10 manuscripts met the basic criteria for data extraction by expressing clear research questions with adequate data for exploration. However, the participants in 8 studies were not representative of the general target population (23, 25–29, 31, 32); 3 of these manuscripts utilized the same single-institution cancer registry (25–27). Importantly, all 8 of the quantitative non-randomized studies utilized appropriate measurements for both their independent and dependent variables. However, there was not sufficient information to determine if outcome data were complete for Chen et al. (25, 26) or Garcia et al. (28). While the former two studies both reported missing biomarker data for many patients, missing values were imputed by chained equations to account for this. Furthermore, while examining mortality, participants in these two studies were inferred to be alive at the date of loss to follow-up, potentially limiting completeness of this outcome as well. For Garcia et al., variable sample sizes were utilized within certain statistical analyses without explicitly reporting on missing data, thus there was not sufficient information to make a ruling on completeness. For most quantitative non-randomized studies, confounders were adequately considered, and exposures occurred as intended. Overall, concern for bias remained low across this cohort of publications (full MMAT results documented in Supplementary Table 2).

Following data extraction, these 10 studies were broadly divided into 2 major categorical themes related to surgical outcomes: (a) AL as a specific predictor of surgical outcomes and (b) AL as an explanatory mechanism for disparities in surgical populations. A third unrelated grouping focused on the utility of specific unique markers of AL among surgical populations. Overlapping themes among the articles were observed.

### Measurement of AL as a dependent Variable

Of the 10 studies included, 8 utilized biomarkers to operationalize AL (Table 2). Among those, 3 utilized an identical multi-system approach to create a composite AL index score for each individual study participant (25–27). Chen et al. (25–27) utilized the combination of 10 measures encompassing multiple physiologic domains, including heart rate, systolic blood pressure, diastolic blood pressure, BMI, alkaline phosphatase, blood glucose, albumin, WBC count, creatinine, and BUN. To create composite scores, each biomarker result falling within the least favorable quartile was assigned 1 point, with a total of 10 points representing the maximum composite AL score. Any score above 2.0—the median for the AL study cohorts—was characterized as high (vs. low ≤ 2.0).

**Table 2:** Biomarkers utilized across quantitative allostatic load and surgery studies.

Authorship	Physiologic indicators of stress	Physiologic systems represented	Composite AL index calculated
Abshire et al., 2018 (23)	Salivary cortisol, salivary CRP, sleep quality (via PSQI) ***(3 total measures)***	Endocrine ***(1 system)***	No
Chen et al., 2024 (25)	HR, SBP, DBP, BMI, alkaline phosphatase, blood glucose, albumin, WBC, BUN, creatinine ***(10 total measures)***	Cardiovascular Metabolic Immunologic Renal ***(4 total systems)***	Yes*
Chen et al., 2024 (26)	HR, SBP, DBP, BMI, alkaline phosphatase, blood glucose, albumin, WBC, BUN, creatinine ***(10 total measures)***	Cardiovascular Metabolic Immunologic Renal ***(4 total systems)***	Yes*
Chen et al., 2024 (27)	HR, SBP, DBP, BMI, alkaline phosphatase, blood glucose, albumin, WBC, BUN, creatinine ***(10 total measures)***	Cardiovascular Metabolic Immunologic Renal ***(4 total systems)***	Yes*
Garcia et al., 2021 (28)	Serum IL-6, CRP, estrogen, free estradiol, salivary cortisol ***(5 total measures)***	Immunologic Endocrine ***(2 total systems)***	No
Hong et al., 2022 (29)	TSH, free T4, T3 ***(3 total measures)***	Endocrine ***(1 system)***	No
Irelli et al., 2022 (31)	Comorbidity variables (via CIRS, perceived pain index ***(2 total measures)***	Cardiac Vascular Hematologic Respiratory Ophthalmological/otolaryngologic Upper GI system Lower GI system Hepatic and pancreatic Renal GU Musculoskeletal/dermatological Neurological Endocrine (metabolic) Psychiatric ***(14 total systems)***	No
McGuinness et al., 2018 (32)	Molecular transcripts, serum creatinine ***(2 total measures)***	Renal ***(1 system)***	No

BMI, body mass index; BUN, blood urea nitrogen; CIRS, chronic inflammatory response syndrome; CRP, C-reactive protein; DBP, diastolic blood pressure; IL-6, interleukin-6, PSQI, Pittsburgh sleep quality index; SBP, systolic blood pressure; TSH, thyroid-stimulating hormone; WBC, white blood cell count.

* Distributions of each biomarker were calculated and combined into a composite AL score ranging from 0 to 10. Composite scores were then dichotomized into high and low AL using the cohort's median score (2.0) as the cutoff.

Although the remaining 5 of 8 studies did not estimate composite AL scores, they did utilize a variety of indicators to quantify the physiologic adaptation to stress. Specifically, Garcia et al. examined a combination of 5 measures: IL-6, CRP, estrogen, free estradiol, and salivary cortisol (28). Similarly, Abshire et al. obtained salivary CRP and salivary cortisol levels (23). Hong & Lee examined thyroid function tests, specifically TSH, free T4, and T3 (29). With a specific focus on renal transplants, McGuinness examined molecular signaling transcripts from tissue biopsies, whereas Irelli et al. employed a broader approach via the Cumulative Illness Rating Scale (CIRS) as a proxy to assess AL over the span of 14 physiologic systems (31, 32). These did not share any overlapping measures with that of the multiple Chen et al. studies described above, illustrating the variety in which AL is quantified as either an outcome or exposure. Of note, although Becker & Granzotti did not attempt to calculate AL in their retrospective cohort study, the potential to collect biomarkers spanning multiple body systems was discussed (24).

### Association between AL and specific surgical outcomes

AL was examined as a driver of surgical outcomes across a broad array of surgical specialties. Abshire et al. explored the relationship between AL and both left ventricular assist device (LVAD) implant purpose—bridge-to-transplant (BTT) vs. destination therapy (DT)—and quality of life (QoL)/functional status (23). The authors found that aside from elevated waking cortisol in the DT group, there were no significant differences in AL markers nor perceived stress, depression, or fatigue between the groups. Higher salivary cortisol as measured by the area under the curve (AUC) were associated with improved postoperative functional status but not QoL. Conversely, poor sleep quality and increased psychologic stress response were related to lower QoL.

Chen et al. (27) focused on surgical clinical outcomes, reporting associations between high AL and incidence of post-operative complications (POC) among women surgically treated for breast cancer. Specifically, the authors demonstrated a linear dose-response relationship between increased AL and development of POC. Continuing the topic of surgical oncology, Chen et al. (25) found mortality risk to be greatest in breast cancer patients with high AL who live in low opportunity neighborhoods—although this did not apply to those with high AL in higher-opportunity areas, emphasizing the contributions of other social determinants of health to surgical outcomes.

Exploring outcomes of initial surgical revascularization procedures in adults with severe peripheral arterial disease (PAD) in relation to ZIP code-level median household income (MHI), Hughes et al. found that increasing MHI was inversely related to patients requiring subsequent amputation and that 4th (vs. 1st) MHI quartile patients had significantly lower 30-day readmission rates (30). AL in the form of chronic biologic adaptation was discussed as a potential driver for disparity in PAD severity, thereby increasing the risk of adverse surgical outcomes in these patients.

Lastly, McGuinness et al. explored the relation between AL and surgical outcomes at the molecular level, identifying specific epigenetic and signaling alterations that correspond with AL and delayed graft function of transplanted kidneys, which is an important postoperative outcome in transplant surgery (32).

### AL as a mechanistic contributor to racial and socioeconomic disparities in surgical outcomes

Across the literature, AL has been examined in the context of a wide variety of health disparities. We found in our review that AL was also specifically hypothesized as a mechanism for health disparities in surgical populations. Becker & Granzotti, utilizing the Healthcare Utilization Project-Nationwide Inpatient Sample (HCUP-NIS), found females and Black males to have higher in-hospital coronary artery bypass grafting mortality rates (24). The authors proposed that mitigating AL among Black patients could reduce this racial disparity. With a focus on AL and mortality among patients surgically treated for breast cancer, Chen et al. (25, 26) explored the influence of neighborhood opportunity and racialized economic segregation, respectively. They found that low neighborhood opportunity was associated with a greater risk for all-cause mortality and that living in neighborhoods with high racialized economic segregation was likewise associated with increased risk of all-cause mortality (25, 26). These were both associated with high AL, which was proposed as a mediator of the observed relationship between living in a segregated environment and all-cause mortality. Lastly, Hughes et al., as referenced above, posed AL and the “biology of poverty” as being mechanistic driver of PAD severity and corresponding surgical bypass failure (30).

### Unique predictors of AL for surgical populations

Among these original studies, 4 specifically highlighted the potential utility of unique biomarkers as predictors of AL among surgical populations in certain clinical contexts. In relation to the 10 biomarkers collected as part of the standard pre-operative evaluation for breast cancer surgery, Chen et al. (27) identified albumin as a primary measure associated with development of POC. In patients undergoing thyroidectomy, Hong & Lee found TSH to increase with the occurrence of stressful events as determined by a modified version of the Stress Overload Scale-Short (SOSS) questionnaire (29). In a broader approach, Irelli et al. demonstrated the utility of the CIRS as a measure of physical impairment among patients with breast cancer (31). As physical impairment was predictive of negative emotional adaptation, this was posed as a component of AL that could be adopted as a screening tool to facilitate targeted psychological support. Finally, Garcia et al. (2021) described loneliness, as characterized by the UCLA Loneliness Scale, as a potential correlate with AL in certain patient populations (28). Specifically, among adult female patients diagnosed with Chiari malformation type I status-post surgical decompression, increased loneliness was associated with increased cortisol levels, which are known to represent aspects of the physiologic stress response. Of note, this relationship only existed among the patients who had undergone surgical decompression.

### Additional findings from reviews and editorials

Given the limited number of original studies that met our full inclusion criteria, additional relevant reviews and author commentaries were carefully examined to generate a more robust understanding of the association between AL and surgery. Through the screening process, 9 relevant reviews and author commentaries were identified (33–41). Collectively, these publications reinforced the themes identified above—including AL measurement, its role in explaining health disparities, and its potential in preoperative risk assessment. Askay & Patterson highlighted delayed wound healing as a response to psychologic stress in surgical patients, and Caumo et al. argued that the brief measure of emotional preoperative stress (B-MEPS) should be used as a tool to inform interventions (i.e., preoperative relaxation, sedation, music therapy, or biofeedback) to improve postoperative outcomes (33, 34). Key findings across these selected articles are noted in Supplementary Table 3.

## Discussion

This systematic review represents the first comprehensive synthesis of literature surrounding AL in surgical care. Our study identified 10 original research manuscripts examining this physiologic phenomenon in the context of surgical outcomes. Taken altogether, the findings highlighted across these articles suggest AL is not only associated with adverse surgical outcomes, but also may be used to account for racial and socioeconomic disparities that continue to occur in surgical populations. As the scope of nearly half of our included studies relates to patients with breast cancer, AL appears to have a measurable impact in the field of surgical oncology in particular—which likely also applies to other complex surgical specialties.

Importantly, AL emerged as a clear hypothesis for why disparities persist among surgical patients based on racial identity and other measures of social vulnerability. When exploring potential explanations for the worsening PAD severity underlying surgical revascularization failure among those with lower median household incomes, Hughes et al. referenced the concept of the “biology of poverty” as a fundamental catalyst (30). First proposed as a theoretical framework by McEwen & Stellar in 1993 and later transformed to a measurable phenomenon in 2001 by Seeman et al., AL has long been recognized as the physiologic consequence of many social determinants of health, including living in a high-deprivation neighborhood, lack of social support, and racial minority status in a racialized society entrenched in systemic racism (10, 42). As the literature has expanded, the terms “biology of poverty”, “weathering”, and “allostatic load” have all coalesced around the shared principle that chronic exposure to social and economic stress contributes to adverse health outcomes, especially among racial minority groups (43). Our findings, especially those exploring neighborhood opportunity and racialized economic segregation, AL, and all-cause mortality among patients undergoing surgery, demonstrate that AL as an explanatory mechanism for disparate outcomes now extends to the surgical sphere (24–26).

Extrapolating from the results described in this review, AL has the potential to identify high-risk surgical patients who may especially benefit from targeted interventions to mitigate their physiologic response to AL. This is particularly critical given that several publications in recent years have found that AL can be reduced through specific lifestyle modifications and therapeutic strategies (44). Cognitive behavioral therapy (CBT), tai chi chih, and cancer-based support groups have specifically been found to decrease AL when used in their appropriate contexts, and multiple studies using the National Health and Nutrition Examination Survey (NHANES) established inverse relationships with AL and both vigorous leisure-time physical activity and dietary quality (44–47). Notably, these have yet to be studied in surgical populations. If standard perioperative optimization strategies (i.e., prehabilitation, nutritional counseling, and stress reduction) are combined with reliable AL quantification, AL-informed patient counseling could play a role in mitigating adverse surgical outcomes—from improving quality of life to possibly even reducing mortality risk (23, 25, 48). This is especially pertinent to oncology patients receiving care at comprehensive cancer centers, where psychosocial and patient support services are already viewed as vital components of a holistic treatment approach (49).

Given the clinical relevance of AL, a standardized approach to its reliable measurement is necessary. Our study identifies a variety of methods used to operationalize this biologic concept as either a dependent variable or covariate. The authorship groups led by J.C. Chen and S. Obeng-Gyasi demonstrated the greatest consistency across 3 studies, calculating a composite AL score for each study participant by evaluating 10 total measures spanning four physiologic systems (25–27). Although alternative approaches in additional included studies measured TFTs, salivary cortisol, or even molecular transcripts, the multi-system approach noted above is most consistent with that first established by Seeman et al. (10, 50). In their foundational work, Seeman et al. measured biomarkers spanning cardiovascular (systolic and diastolic blood pressure), metabolic (waist-hip ratio, HDL cholesterol, total cholesterol, HbA1c), and neuroendocrine (DHEA-S, urinary cortisol, norepinephrine, and epinephrine levels) systems. More recently, Chyu & Upchurch employed a slightly different set of 10 biomarkers, including systolic and diastolic blood pressure, resting pulse rate, homocysteine, CRP, serum albumin, HbA1c, HDL, total cholesterol, and BMI (11). Although the consistent methodology of J.C. Chen and S. Obeng-Gyasi represents a strong attempt at standardizing AL assessment of surgical populations—especially as these values can all be obtained through routine preoperative care, which often includes the collection of a complete blood count (CBC) and comprehensive metabolic panel (CMP)—further consensus is needed on the optimal strategy for measuring AL in the perioperative setting. Establishing a standardized surgical AL index would not only facilitate clinical utilization of AL as a predictor for surgical risk, but also enable more robust comparisons across studies exploring this concept and potential interventions that counter its effects.

Beyond the findings identified and discussed above, several original studies published after our systematic review was conducted further substantiate and extend our findings. Specifically, these publications extended the scope of AL research to other realms of surgical oncology, including hepatopancreatobiliary (HPB) cancer surgery, colorectal cancer surgery, and gynecologic oncology (7, 51, 52). Among patients who underwent HPB cancer surgery, high AL was associated with elevated risk of both Clavien-Dindo grade IV complications and mortality (52). For patients undergoing surgery for colorectal cancer, high AL was likewise associated with increased odds of postoperative complications, extended length of hospital stays, and perioperative mortality (51). Lastly, among patients with epithelial ovarian cancer, AL was associated with significantly elevated mortality risk, even after adjusting for clinical, demographic, and treatment-related factors (7). Notably, all three of these investigations utilized the same 10 biomarkers as reported by J.C. Chen and S. Obeng-Gyasi to estimate AL. These recent studies only further emphasize the magnitude that this physiologic phenomenon has within the field of surgical oncology, spanning multiple diseases and procedures.

This systematic review has limitations to note. Despite expanding our initial literature search to 5 large databases, only 10 original studies met our full inclusion criteria. The breadth of available literature may have been limited by publication bias, as studies with null findings related to AL and surgical outcomes may be underrepresented. Among the 10 included publications, not all directly quantified AL using specific biomarkers. Furthermore, among those authors who did attempt to quantify AL, considerable heterogeneity existed in measurement, creating inherent challenges in directly comparing methodology and outcomes. Additionally, the types of procedures represented were mostly pertinent to surgical oncology; a wider array of surgical specialties with discussion of more generalized perioperative complications would be informative. As identified within the MMAT quality and bias assessment, the majority of all studies that met our inclusion criteria were ultimately found not to have samples representative of their broader target populations. Lastly, the predominance of retrospective single-institution studies limits generalizability and the ability to conclude cause-and-effect relationships within their findings. This points to an important area of opportunity for future research where large, multi-institution databases with demographic diversity—in addition to prospective work—should be employed to increase the external validity and ability to more fully interpret those results. Even with these limitations, this review benefits from a rigorous multi-database search strategy and adherence to PRISMA guidelines, providing a reliable foundation for future investigation.

In conclusion, this systematic review provides a comprehensive evaluation of the growing body of literature exploring the association between AL and surgical outcomes, thereby establishing a vital academic framework for how these relationships may be viewed, discussed, and subsequently investigated in future studies. By illuminating the connections between AL and surgery, targeted interventions aimed at reducing AL may be implemented to mitigate postoperative complications and improve the quality of surgical care for all patients, including those who are most socially vulnerable and therefore subjected to higher AL. Surgical oncology currently appears to be leading the charge on AL research in the field of surgery, and this burgeoning area of research has the potential to uncover important opportunities to mitigate the effects of environmental stress in this population. Future work in this area should include prospective studies examining preoperative AL, specific postoperative outcomes, and the impact of personalized AL-centered interventions on adverse perioperative outcomes. Emphasis should also be put towards formally establishing a consensus for a feasible, accurate approach in calculating AL, thereby mitigating the heterogeneity observed across prior studies.

## Data Availability

The original contributions presented in the study are included in the article/Supplementary Material, further inquiries can be directed to the corresponding author.
