# Supplementary material for: The effects of allostatic load on patients undergoing surgery: a systematic review
Source: Front Surg. 2026 Aug 21;13:1866869. doi: 10.3389/fsurg.2026.1866869 (PMC13541757; doi:10.3389/fsurg.2026.1866869)
Supplement: Supplementary file 1 [file Table1.docx]

**Supplementary Table 1. Search Queries Translated Across Literature Databases**

| **Literature Database** | **Search Query** |
| --- | --- |
| PubMed | ("Allostasis"[Mesh] OR "allosta*"[tiab] OR "Socioenvironmental stressor*"[tiab] OR “Socioenvironmental factor*”[tiab]) AND ("Surgical Procedures, Operative"[Mesh] OR "Postoperative Complications"[Mesh] OR "surgi*"[tiab] OR "surger*"[tiab] OR “postsurg*”[tiab] OR “post-surg*”[tiab] OR “postoperative*”[tiab] OR "post-operative*"[tiab]) |
| Embase | ('allostasis'/exp OR 'allostatic load'/exp OR 'allostatic load index'/exp OR 'allostatic overload'/exp OR 'allosta*':ti,ab,kw OR 'socioenvironmental stressor*':ti,ab,kw OR 'socioenvironmental factor*':ti,ab,kw) AND ('surgery'/exp OR 'postoperative complication'/exp OR 'surgi*':ti,ab,kw OR 'surger*':ti,ab,kw OR ‘postsurg*’:ti,ab,kw OR 'post surg*':ti,ab,kw OR ‘postoperative*’:ti,ab,kw OR 'post-operative*':ti,ab,kw) NOT ('animal'/exp NOT 'human'/exp) |
| Cochrane | 1. [mh "Allostasis"]  2. allosta*:ti,ab OR "Socioenvironmental stressor":ti,ab OR "Socioenvironmental stressors":ti,ab OR “Socioenvironmental factor”:ti,ab OR “Socioenvironmental factors”:ti,ab 3. #1 OR #2 4. [mh "Surgical Procedures, Operative"] OR [mh "Postoperative Complications"] 5. surgi*:ti,ab OR surger*:ti,ab OR postsurg*:ti,ab OR post-surg*:ti,ab OR postoperative*:ti,ab OR post-operative*:ti,ab 6. #4 OR #5 7. #3 AND #6 |
| Web of Science | TS=("allosta*" OR "Socioenvironmental stressor*" OR “Socioenvironmental factor*”) AND TS=("surgi*" OR "surger*" OR “postsurg*” OR “post-surg*” OR “postoperative*” OR "post-operative*") |
| PsychINFO | S1 AND S2  S1 (Surgery) (DE "Surgery" OR DE "Amputation" OR DE "Bariatric Surgery" OR DE "Circumcision" OR DE "Cochlear Implants" OR DE "Colostomy" OR DE "Endocrine Gland Surgery" OR DE "Heart Surgery" OR DE "Hysterectomy" OR DE "Neurosurgery" OR DE "Organ Transplantation" OR DE "Ovariectomy" OR DE "Plastic Surgery" OR DE "Postsurgical Complications" OR DE "Sterilization (Sex)" OR DE "Transection" OR DE "Surgical Patients") OR TI("surgi*" OR "surger*" OR postsurg* OR post-surg* OR postoperative* OR post-operative*) OR AB("surgi*" OR "surger*" OR postsurg* OR post-surg* OR postoperative* OR post-operative*) OR KW("surgi*" OR "surger*" OR postsurg* OR post-surg* OR postoperative* OR post-operative*)  S2 (Allostasis) KW("allosta*" OR "Socioenvironmental stressor*" OR “Socioenvironmental factor*”) OR TI("allosta*" OR "Socioenvironmental stressor*" OR “Socioenvironmental factor*”) OR AB("allosta*" OR "Socioenvironmental stressor*" OR “Socioenvironmental factor*”) |

**Supplementary Table 2. MMAT Assessment of Quality and Bias Across Included Studies**

| **Authorship** | **Study Design** | | **Screening** | | | **Quantitative**  **Non-Randomized** | | | | | | | | | | **Quantitative Descriptive** | | | | | | | | | | **Total Score** | |  |
| --- | --- | --- | --- | --- | --- | --- | --- | --- | --- | --- | --- | --- | --- | --- | --- | --- | --- | --- | --- | --- | --- | --- | --- | --- | --- | --- | --- | --- |
|  |  | | **S1** | | **S2** | **3.1** | | **3.2** | | **3.3** | | **3.4** | | **3.5** | | **4.1** | | **4.2** | | **4.3** | | **4.4** | | **4.5** | |  | |  |
| Abshire et al., 2018 (23) | Quantitative Descriptive (Cat. 4) | | Yes | | Yes |  | |  | |  | |  | |  | | Y | | N | | Y | | N | | Y | | 3/5 | |  |
| Becker & Granzotti, 2019 (24) | Quantitative Non-Randomized (Cat.3) | | Yes | | Yes | Y | | Y | | Y | | Y | | Y | |  | |  | |  | |  | |  | | 5/5 | |  |
| Chen et al., 2024 (25) | Quantitative Non-Randomized (Cat.3) | | Yes | | Yes | N | | Y | | C | | Y | | Y | |  | |  | |  | |  | |  | | 3/5 | |  |
| Chen et al., 2024 (26) | Quantitative Non-Randomized (Cat.3) | | Yes | | Yes | N | | Y | | C | | Y | | Y | |  | |  | |  | |  | |  | | 3/5 | |  |
| Chen et al., 2024 (27) | Quantitative Non-Randomized (Cat.3) | | Yes | | Yes | N | | Y | | Y | | Y | | Y | |  | |  | |  | |  | |  | | 4/5 | |  |
| Garcia et al., 2021 (28) | Quantitative Non-Randomized (Cat.3) | | Yes | | Yes | N | | Y | | C | | N | | C | |  | |  | |  | |  | |  | | 1/5 | |  |
| Hong & Lee, 2022 (29) | Quantitative Non-Randomized (Cat.3) | | Yes | | Yes | C | | Y | | Y | | Y | | C | |  | |  | |  | |  | |  | | 3/5 | |  |
| Hughes et al., 2022 (30) | Quantitative Non-Randomized (Cat.3) | | Yes | | Yes | Y | | Y | | Y | | Y | | Y | |  | |  | |  | |  | |  | | 5/5 | |  |
| Irelli et al., 2022 (31) | Quantitative Non-Randomized (Cat.3) | | Yes | | Yes | N | | Y | | Y | | N | | Y | |  | |  | |  | |  | |  | | 3/5 | |  |
| McGuinness et al., 2018 (32) | Quantitative Descriptive (Cat. 4) | | Yes | | Yes |  | |  | |  | |  | |  | | Y | | N | | Y | | C | | Y | | 3/5 | |  |
| *Y – Yes, C – Can't tell, N – No* | |  | |  |  | |  | |  | |  | |  | |  | |  | |  | |  | |  | |  | |  | |

**Supplementary Table 3. Results from Relevant Reviews of Allostatic Load and Surgical Concepts**

| **Authorship** | **Findings Pertaining to Allostatic Load** |
| --- | --- |
| Askay & Patterson, 2008 (33) | • Wound healing noted to be slower under psychologic stress (i.e., pain) • Reported findings from outside study that higher post-surgical pain levels delayed wound healing from a punch biopsy in women status-post gastric bypass surgery, even after controlling for pre-surgery depressive symptoms and other post-surgical medical complications |
| Caumo et al., 2017 (34)* | • B-MEPS items allow the identification of patients with a higher vulnerability to environmental stressors • Proposed that B-MEPS scores can help plan specific interventions (i.e. preoperative relaxation, sedation, music therapy, biofeedback) to improve postoperative outcomes • Used model of AL to explain preoperative emotional stress as a potential risk factor for postoperative pain |
| Chen et al., 2024 (35)** | • Biomarkers used in Chen et al. (26) were components of routine preoperative work-up • Additional research is needed to the understand mechanism for how neighborhood factors impact the influence of AL on oncologic outcomes |
| Chong et al., 2015 (36) | • Mechanisms of AL resulting from acute and chronic stress described • Cites Seeman et al. (53) for establishing an inverse relationship between SES and AL using 16 biomarkers spanning cardiovascular, respiratory, renal, inflammatory, endocrine, and metabolic systems • Hierarchic logistic regression revealed that AL outperforms comorbidities as predictor of mortality • Proposes mechanism that social disadvantages reversibly alters epigenetics and causes aberrant stres response, increasing sensitivity to operative stress • Argues that SES should be incorporated into surgical risk assessment |
| Christodoulou, 2017 (37) | • Reported that no article explored AL as consequence of surgical approach • Cited Chong et al. (36) as the only article connecting surgery to allostasis • Kynurenine levels may be used to assess AL and predict risk of surgical complications • Surgical approach (minimally invasive/robotic surgery) may be a consideration in reducing operative stress, though this has not been evaluated using biomarkers |
| Hughes & Sehgal, 2018 (38) | • Black (vs white) patients are more likely to undergo a major lower extremity amputation • Racial/ethnic disparities in cardiovascular disease exist even after controlling for SES • Proposes that AL (in relation to the concept of weathering/biology of poverty) contributes to these disparities • Cited Kalinowski et al. (54) to suggest that increased levels of chronic inflammation may mediate the greater prevalence of PAD in Black patients |
| Katon et al., 2023 (39) | • Prevalence of uterine fibroids is higher in Black (vs white) women • Cites multiple studies showing association between chronic psychologic stress/racism and increase risk of fibroids, especially among Black women • Black (vs white) women with fibroids or endometriosis experience worse surgical outcomes (i.e. surgical complications, longer surgery times, or readmission) independent of surgical approach • AL proposed as mechanism for racial disparities in fibroids and endometriosis, impacting incidence, severity, and/or access to care |
| Vogel, 2012 (40) | • Cites multiple studies showing higher incidence of post-operative sepsis in Black (vs white) patients • Cites Chyu & Upchurch (11) for calculating AL via 10 biomarkers spanning cardiovascular, inflammatory, and metabolic system and for identifying Black females as having the highest AL scores • AL proposed as mechanism contributing to this racial disparity in surgical sepsis |
| Wynn & Holloway, 2019 (41) | • Chronic stress is associated with increases in inflammatory cytokines that prolong wound healing • Noted that patient perceptions of stress may not adequately predict risks posed by AL on wound healing  • Identifies a need for both qualitative and quantitative measures to assess the impact of stress on healing • Suggests AL may serve as reliable measure by utilizing biomarkers (i.e. cortisol, cholesterol, blood pressure, BMI) |
| **Author reply **Author reflection* |  |

**References:**

11. Chyu L, Upchurch DM. Racial and Ethnic Patterns of Allostatic Load Among Adult Women in the United States: Findings from the National Health and Nutrition Examination Survey 1999–2004. J Womens Health. 2011 Apr;20(4):575–83. doi:10.1089/jwh.2010.2170

23. Abshire M, Bidwell JT, Page G, Budhathoki C, Davidson PM, Russell SD, et al. Physiological and Psychological Stress in Patients Living With a Left Ventricular Assist Device. ASAIO J. 2018 Nov;64(6):e172–80. doi:10.1097/MAT.0000000000000847

24.Becker ER, Granzotti AM. Trends in In-hospital Coronary Artery Bypass Surgery Mortality by Gender and Race/Ethnicity --1998-2015: Why Do the Differences Remain? J Natl Med Assoc. 2019 Oct;111(5):527–39. doi:10.1016/j.jnma.2019.04.009

25. Chen JC, Elsaid MI, Handley D, Plascak JJ, Andersen BL, Carson WE, et al. Association Between Neighborhood Opportunity, Allostatic Load, and All-Cause Mortality in Patients With Breast Cancer. J Clin Oncol. 2024 May 20;42(15):1788–98. doi:10.1200/JCO.23.00907

26. Chen JC, Handley D, Elsaid MI, Plascak JJ, Andersen BL, Carson WE, et al. The Implications of Racialized Economic Segregation and Allostatic Load on Mortality in Patients with Breast Cancer. Ann Surg Oncol. 2024 Jan;31(1):365–75. doi:10.1245/s10434-023-14431-1

27. Chen JC, Elsaid MI, Handley D, Anderson L, Andersen BL, Carson WE, et al. Allostatic load as a predictor of postoperative complications in patients with breast cancer. Npj Breast Cancer. 2024 Jun 12;10(1):44. doi:10.1038/s41523-024-00654-2

28. Garcia MA, Li X, Allen PA, Delahanty DL, Eppelheimer MS, Houston JR, et al. Impact of Surgical Status, Loneliness, and Disability on Interleukin 6, C-Reactive Protein, Cortisol, and Estrogen in Females with Symptomatic Type I Chiari Malformation. The Cerebellum. 2021 Dec;20(6):872–86. doi:10.1007/s12311-021-01251-w

29. Hong H, Lee J. Thyroid-Stimulating Hormone as a Biomarker for Stress After Thyroid Surgery: A Prospective Cohort Study. Med Sci Monit. 2022 Oct 20;28. doi:10.12659/MSM.937957

30. Hughes K, Olufajo OA, White K, Roby DH, Fryer CS, Wright JL, et al. The influence of socioeconomic status on outcomes of lower extremity arterial reconstruction. J Vasc Surg. 2022 Jan;75(1):168–76. doi:10.1016/j.jvs.2021.08.071

31. Irelli A, Ranieri J, Sirufo MM, De Pietro F, Casalena P, Ginaldi L, et al. Allostatic Load as an Insight into the Psychological Burden after Primary Treatment in Women with Breast Cancer: Influence of Physical Side Effects and Pain Perception. J Clin Med. 2022 Apr 12;11(8):2144. doi:10.3390/jcm11082144

32. McGuinness D, Mohammed S, Monaghan L, Wilson PA, Kingsmore DB, Shapter O, et al. A molecular signature for delayed graft function. Aging Cell. 2018 Oct;17(5):e12825. doi:10.1111/acel.12825

33. Askay SW, Patterson DR. What are the psychiatric sequelae of burn pain? Curr Pain Headache Rep. 2008 Apr;12(2):94–7. doi:10.1007/s11916-008-0018-1

34. Caumo W, Segabinazi JD, Stefani LPC. Reply: allostatic load as an approach to support the theoretical assumptions of the Brief Measure of Emotional Preoperative Stress (B-MEPS). Br J Anaesth. 2017 Apr;118(4):638–40. doi:10.1093/bja/aex064

35. Chen JC, Pawlik TM, Obeng-Gyasi S. ASO Author Reflections: Racialized Economic Segregation and Allostatic Load: The Impact of Internalizing Our Residential Environments. Ann Surg Oncol. 2024 Feb;31(2):1005–6. doi:10.1245/s10434-023-14507-y

36. Chong V, Imhoff L, Harken A. Socioeconomic status and the assessment of operative risk. Surgery. 2015 Apr;157(4):605–8. doi:10.1016/j.surg.2014.09.032

37. Christodoulou I. The allostatic load caused by surgery. Vol. 20. 2017;20(2):149–53.

38. Hughes K, Sehgal N. Racial/ethnic Disparities in Lower Extremity Amputation Vs Revascularization: A Brief Review. J Natl Med Assoc. 2018 Dec;110(6):560–3. doi:10.1016/j.jnma.2018.02.003

39. Katon JG, Plowden TC, Marsh EE. Racial disparities in uterine fibroids and endometriosis: a systematic review and application of social, structural, and political context. Fertil Steril. 2023 Mar;119(3):355–63. doi:10.1016/j.fertnstert.2023.01.022

40. Vogel TR. Update and Review of Racial Disparities in Sepsis. Surg Infect. 2012 Aug;13(4):203–8. doi:10.1089/sur.2012.124

41. Wynn M, Holloway S. The impact of psychological stress on wound healing: a theoretical and clinical perspective. Vol. 15. 2019;15(3).

53. Seeman TE, Crimmins E, Huang MH, Singer B, Bucur A, Gruenewald T, et al. Cumulative biological risk and socio-economic differences in mortality: MacArthur Studies of Successful Aging. Soc Sci Med. 2004 May;58(10):1985–97. doi:10.1016/S0277-9536(03)00402-7

54. Kalinowski L, Dobrucki IT, Malinski T. Race-Specific Differences in Endothelial Function: Predisposition of African Americans to Vascular Diseases. Circulation. 2004 Jun;109(21):2511–7. doi:10.1161/01.CIR.0000129087.81352.7A
